# Implementation of a Stepwise, Multidisciplinary Intervention for Pain and Challenging Behaviour in Dementia (STA OP!): A Process Evaluation

**DOI:** 10.5334/ijic.3973

**Published:** 2018-09-07

**Authors:** Marjoleine J. C. Pieper, Wilco P. Achterberg, Jenny T. van der Steen, Anneke L. Francke

**Affiliations:** 1 Amsterdam Public Health (APH) research institute, van der Boechorststraat 7, 1081 BT Amsterdam, NL; 2 Department of General Practice & Elderly Care Medicine, VU University Medical Center Amsterdam, van der Boechorststraat 7, 1081 BT Amsterdam, NL; 3 Department of Public Health and Primary Care, Leiden University Medical Center, Post zone V0-P, 2300 RC Leiden, NL; 4 Department of Public and Occupational Health, VU University Medical Center Amsterdam, van der Boechorststraat 7, 1081 BT Amsterdam, NL; 5 Netherlands Institute for Health Services Research (NIVEL), 3500 BN Utrecht, NL

**Keywords:** behaviour, dementia, Health Plan Implementation, intervention, nursing homes, pain, program evaluation, process evaluation

## Abstract

**Background:**

A stepwise, multidisciplinary and multicomponent intervention (called STA OP!) was implemented in Dutch nursing home units, which included a comprehensive multidisciplinary team training. A cluster-randomised controlled trial showed that the intervention reduced symptoms of pain and challenging behaviour.

**Objective(s):**

To describe the experiences around the implementation of the intervention; to examine the extent to which the STA OP! intervention was delivered and implemented as intended (at the level of the team, and the individual resident/professional); and to understand factors influencing the implementation process.

**Methods:**

A process evaluation was performed using a mixed-methods design encompassing several data sources. Quantitative data (i.e. from the written evaluations by healthcare professionals, management, and the research database) were analysed using descriptive statistics. Qualitative data (i.e. semi-structured interviews, notes, completed intervention forms, and written evaluations) were analysed according to the principles of thematic analysis. The implementation process and the influencing factors were categorised according to the i) organisational level, ii) the team level, and iii) the level of the individual resident/professional.

**Results:**

In total, 39.2% of the residents with pain and/or challenging behaviour were treated following the stepwise approach of the STA OP! intervention. The training manual and forms used were found to be relevant and feasible. Factors inhibiting the implementation process at the i) organisational level concerned instability of the organisation and the team (e.g. involvement in multiple projects/new innovations, staff turnover/absence of essential disciplines, and/or high workload). At the team level (ii), we found that presence of a person with a motivational leadership style facilitated the implementation. Also, interdisciplinary cooperation through the design/setting of the multidisciplinary training, securing the intervention by use of clear agreements, and written reporting or transfers facilitated implementation. At the individual level (iii), perceived value of the stepwise working method, and enhanced awareness facilitated the implementation.

**Conclusion:**

Although the intervention was not implemented as planned, the intervention empowered healthcare professionals and increased their awareness of the signals of pain and challenging behaviour. Future implementation of the intervention should start on units with a motivational leader, and specific features of the organisation and the team should be considered to facilitate implementation, e.g. stability, support, and shared focus to change.

## Highlights

### What is already known about the topic?

- Introducing care innovations remains a challenge since they do not necessarily find their way into practice, even when proven effective and the staff is motivated to use them.
- Barriers for implementation often arise at different levels of the healthcare system, e.g. at the organisational level, team level, and at the level of the individual resident/healthcare professional.

### What this paper adds

- Implementation of a systematic, stepwise intervention for pain and challenging behaviour enhances perceived motivation, awareness and empowerment of healthcare professionals.
- Training an entire multidisciplinary nursing home team facilitates interdisciplinary learning, collaboration and communication.
- Factors inhibiting the implementation process often concern a lack of stability of the organisation and/or the team.
- Factors facilitating the implementation process often concern educational reinforcement, staff engagement, and the presence of a motivational leader.

## Background

Dementia is defined as a ‘clinical syndrome due to disease of the brain, usually of a progressive nature, which leads to disturbances of multiple higher cortical functions, including memory, thinking, orientation, comprehension, calculation, learning capacity, language, and judgment’ [1]. A particular challenge in the care of patients with dementia is the presence of pain. Pain in dementia is often expressed through behavioural disturbances [2,3].

Although both pain and challenging behaviour are highly prevalent in dementia [4], it is the entanglement between the two that makes their relationship, as well as its assessment and treatment, complex and challenging [2,5,6]. To help healthcare professionals deal with these complex problems and challenges, Kovach et al., 2006 developed the Serial Trial Intervention (STI) [7]. The assumption, however, is that knowledge does not suffice [8] (the control group received training targeting knowledge only) and a stepwise working method is needed to change practice.

Because organisation, availability and level of education of the staff, and the availability of additional resources, differ across settings and countries [9,10,11], we translated and adapted the STI [7] for the Dutch language and Dutch nursing home care setting [12]. Psychogeriatric care in Dutch nursing homes is delivered on specialised care units. The nursing staff (i.e. registered nurses, certified nurse assistants, and nurse aides) provide most of the round-the-clock care. Also typical for Dutch nursing homes, is that they employ specialised elderly care physicians to provide medical care. Furthermore, most nursing homes also employ psychologists, physiotherapists and occupational therapists. Together, these professionals form the multidisciplinary care team [11,12,13]. The Dutch version of the STI, called STA OP!, is available for use by the multidisciplinary team [12].

**Figure 1:**
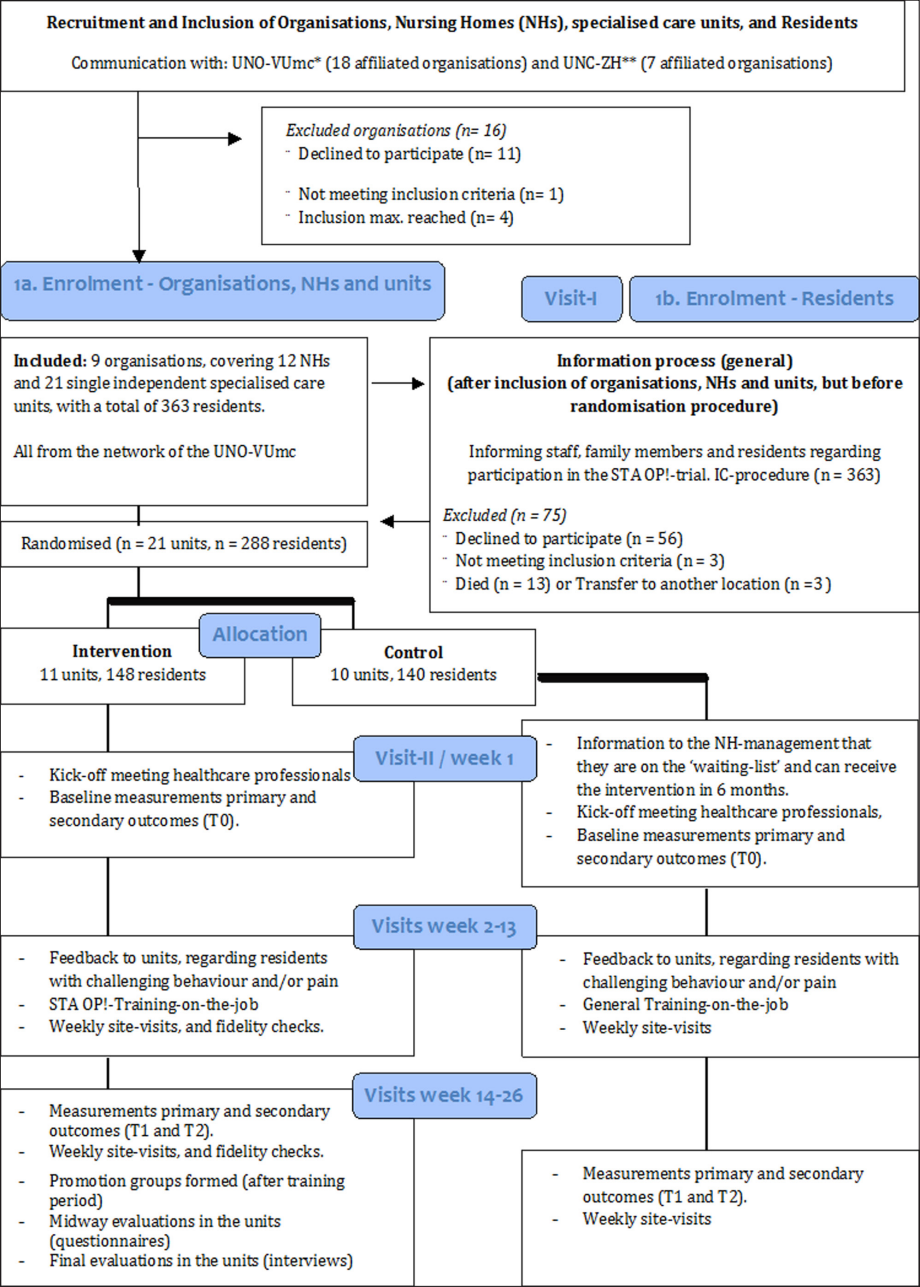
Flowchart of the study design (cluster RCT) and implementation strategies.

However, it is known that care innovations do not automatically find their way into practice, even if staff is motivated to use them. Generally, this requires an active approach and an implementation plan with effective strategies [14,15]. In addition, implementing care innovations (such as the STA OP! intervention) is also challenging because of their complexity i.e. the combination of several interacting components [16] (Figure 1). When studying such complex and multicomponent interventions, an important aspect is whether (or not) the intervention is implemented as planned. Even when it is perfectly designed, ‘real-world’ contextual factors may prevent the intervention from being realized as intended [17,18,19,20,21,22]. Medical Research Council guidance suggests that “Complex interventions may work best if tailored to local circumstances rather than being completely standardised” [18]. Therefore, it is important to investigate how and to what extent the intervention is implemented, and to identify and understand the factors that facilitate or impede implementation, i.e. to gain insight into the implementation process.

Studies of complex interventions have shown that influencing factors can occur at several levels [23,24,25]; the organisational level, the team level, and the individual resident/professional level. For example, some studies [25,26,27,28] described staff turnover, high workload, concurrent projects, and/or organisational changes as barriers for implementing care innovations. The presence of an opinion leader or support of key persons, and a positive attitude towards change, have been described as facilitating factors in the implementation of an intervention [25].

This paper aims to describe the implementation process of the STA OP! intervention. Specific questions addressed are:

1. What are the experiences of healthcare professionals with implementation of the intervention and its actual use in daily practice?
2. Is the intervention delivered and implemented as intended at the level of the team and of the individual resident/professional?
3. In the implementation process, what facilitating or impeding factors are associated with the level of the organisation, the team, or the individual resident/professional?

## Methods

### Design

To describe the process of implementation of the complex intervention and assess barriers and facilitators in an inductive manner, we used a mixed-methods design involving triangulation of researchers, various data sources, and qualitative and quantitative methods. Inspired by the multi-level process evaluation model of Verkaik et al. we classified the factors we had identified at different levels: the organisational level, the team level, and the individual resident/professional level [25] (Table 2).

### Setting and sample

#### Inclusion of organisations and residents

All affiliated organisations of the University Network for Organisations of Elderly care of the VU University Medical Center (UNO-VUmc; 18 organisations) and those affiliated with the University Network of the Care sector-South Holland (UNC-ZH; 7 organisations) were invited to participate in the cluster-randomized controlled trial (RCT) and completed a declaration of intent. Detailed inclusion criteria for organisations and residents are described elsewhere [12], and is presented in a supplementary file.

#### Procedure(s)

Of the 25 eligible organisations, 14 returned a signed declaration of intent and 11 declined to participate because they were already involved in other (research) projects. Subsequently, the project coordinator (MP) and a member of the project team (WA) discussed participation with the management of these 14 organisations in the order in which they arrived: 1 organisation did not meet the inclusion criteria and was excluded; also, because our inclusion maximum was reached, the last 4 organisations reporting for participation were also excluded. This resulted in the final participation of 9 organisations (reach, 64.3%), covering 12 nursing homes with a total of 21 units (Figure 1).

An independent researcher (unaware of the identity of the units) allocated the 21 units to the intervention condition (11 units) or the control condition (10 units), using a computer-generated sequence program [29]. All residents residing on the participating units were invited to participate in the study. Informed consent was provided by the legal representatives of 307 residents (reach 84.6%); 160 residents were enrolled in the intervention condition (52.1%). Due to a transfer to another location (3 residents), or to death (9 residents), the study started with a total of 148 residents (reach, 92.5%) in the intervention condition (Figure 1).

For this process evaluation, only the units in the **intervention condition** are relevant and analysed here.

### Planned implementation strategies & elements of the STA OP! intervention

An implementation strategy combining several components (Figure 1) was pre-defined in the study protocol [12]. Prior to, during and after the implementation of STA OP! various activities were planned at the three organisational levels (Table 2).

#### Multidisciplinary training for healthcare professionals

The STA OP! intervention has a bottom-up organisational style, implying that the nursing team (registered nurses, certified nursing assistants and nurse aides) is ‘in the lead’. Implementation of this intervention at the level of the team was by means of a comprehensive multidisciplinary team training.; all training sessions were offered twice to allow staff to attend because the teams were not paid for replacement of staff during the training. Besides the nursing team, other participants undergoing training were psychologists, elderly care physicians, and occupational therapists/physiotherapists. For each meeting, it was known which disciplines would be required. During a 3-month period (i.e. 5 meetings of 3 h each, every 2–3 weeks) the multidisciplinary team was trained in i) the stepwise working method of the protocol, ii) enhanced physical and affective assessment skills that target the unmet needs commonly found in individuals with advanced dementia (i.e. the STA OP! assessment), and iii) the necessary feedback and communication skills to enhance interdisciplinary communications. In between the meetings, healthcare professionals applied and practised the steps of the intervention in the subgroups formed.

Three experienced trainers with a nursing background (university level) delivered the training sessions. If healthcare professionals attended at least 4 of the 5 meetings they received a certificate.

The actual implementation or utilisation of the intervention occurred at the individual resident level, and started with a ‘behavioural change identification’. A summary of the steps are described elsewhere [12] and presented in Table 1. All healthcare professionals (i.e. a multidisciplinary team) should identify behavioural symptoms using an explicit schedule and procedures. When a resident exhibited a change(s) in behaviour that was not effectively treated, and basic care provided was checked at step 0, the STA OP! was initiated by the registered nurse or certified nursing assistant at step 1. The STA OP! intervention was stopped when the behavioural symptoms decreased or diminished, or if effects were lacking. Continuation with the next steps of the STA OP! was based on the results of the assessments and a decrease in the symptoms within the time frames established for the specified treatments. Ineffective treatments were stopped, and effective treatments were scheduled for regular use and added to the resident’s care plan and therapeutic regimen. If behavioural symptoms continued after completing these 5 steps, the process was repeated at the initial ‘behavioural change identification.’

**Table 1:** Description of the steps of the STA OP! intervention at the individual level according to the protocol [12,30].

Steps	Description

Start with a ‘Behavioural Change Identification’: define the target behaviour, its expression and when (in what situation) this behaviour is challenging. Check if the behaviour is new or recurrent. If the behaviour is recurrent, check what has been done in the past to treat it. Define for whom the behaviour is challenging: the patient, family, or caregivers? A psychologist can be consulted at this step. *If the nurses and the multidisciplinary team of healthcare professionals make a clear description of the targeted behaviour, the nurse moves to the next step (0)*.	

**0**	Perform a basic care needs assessment, and assess if basic care needs are fulfilled (e.g. hunger, thirst, eyeglasses, hearing aids or toileting). *If assessment is positive, a targeted intervention is implemented or the appropriate discipline is consulted to begin treatment. If the assessment is negative, or if treatment fails to decrease symptoms, the nurse moves to the next step (1)*.
**1**	Perform a pain and physical needs assessment. In addition to a brief physical nursing assessment (screening for pain) by the nurse (a), nurses fill out an observational pain instrument (PACSLAC-D) as well (b). This form is handed to the nursing home physician (or if available a nurse practitioner), who performs a more comprehensive physical assessment (c) in order to find other probable physical causes associated with discomfort. For those residents already using pain medication or psychotropic drugs, and still have behavioural symptoms possibly related to pain or affective discomfort, the nursing home physician assesses whether the medication given is in accordance with the guidelines of the World Health Organization (WHO) and Verenso (the Dutch Association of Nursing Home Physicians) (also see steps 4 and 5). *If assessment is positive, a targeted intervention is implemented or the appropriate discipline is consulted to begin treatment. If the assessment is negative, or if treatment fails to decrease symptoms, the nurse moves to the next step (2)*.
**2**	Perform affective needs assessment that focuses on needs of people with dementia: (a) environmental stress threshold not exceeded, (b) balance between sensory-stimulating and sensory-calming activity throughout the day, and (c) receipt of meaningful human interaction each day. The psychologist (or social worker) working in the nursing home can be consulted at this step. *If assessment is positive, a targeted intervention is implemented or the appropriate discipline is consulted to begin treatment. If the assessment is negative, or if treatment fails to decrease symptoms, the nurse moves to the next step (3)*.
**3**	Administer a trial of non-pharmacological comfort treatment(s). Treatments used are customised to the person and the situation, and are based on a list of psychosocial and environmental treatments that are associated with decreasing agitated behaviours. *If a one-time treatment is effective and continued use is desirable, take actions needed to ensure continued treatment (e.g. communicate new treatment to other staff and family, write it down in the patients care plan with prescribed times or administration). If a trial of non-pharmacological comfort treatment(s) does not ameliorate behaviours in a time frame likely to show outcomes, the nurse should move to the next step (4). Stop ineffective treatments*.
**4**	Administer a trial of analgesic agents by either administering the prescribed as-needed analgesic agent or obtaining orders to escalate a current analgesic medication. *If treatment is effective and continued use is desirable, take actions needed to ensure continued treatment (e.g. schedule dosing of effective treatments for continued use, write it down in the patients care plan with prescribed times or administration). If there is not a response to a trial course of analgesic medications, consider consultation regarding further escalation or proceed to the next step (5). Stop ineffective treatments*.
**5**	Consult with other disciplines (e.g. psychiatrist) and/or administer a trial of a prescribed as-needed psychotropic drugs in this step if the behaviour continues and alternatives are carefully considered, and potential side effects are weighs against the comfort needs of the resident. *Monitor for recurrence and new problems. Conduct regular comprehensive assessments. Establish clear criteria for evaluation of problems and treatment effectiveness, need for treatments, and possible side effects. If treatment is negative, and/or behavioural symptoms continue, repeat consultation or the entire process at the initial ‘behavioural change identification’*.

Copyright (2016) Wiley. Used with permission from (Marjoleine J.C. Pieper, Anneke L. Francke, Jenny T. van der Steen, Erik J.A. Scherder, Jos W.R. Twisk, Christine R. Kovach, and Wilco P. Achterberg. Effects of a Stepwise Multidisciplinary Intervention for Challenging Behavior in Advanced Dementia: A Cluster Randomized Controlled Trial. J Am Geriatr Soc., John Wiley & Sons, Inc.).

#### Formation of Core teams

According to the protocol, during the last meeting a core team of 3–4 persons per unit was formed, consisting of a certified nursing assistant or registered nurse, psychologist and elderly care physician and, additionally, an occupational therapist or physiotherapist. The objectives of these core teams were: 1) to facilitate the implementation at the team level, 2) secure the intervention to daily or frequently used internal structures or meetings, and 3) act as a coach regarding problems, questions or queries concerning utilisation of the intervention.

#### Formation of subgroups, and selection of residents

Prior to the first meeting the registered nurse or certified nurse assistant formed subgroups of professionals, consisting of a mixture of disciplines, for educational purposes during the training as well as in clinical practice. Parallel to this, the study coordinator created an overview of eligible residents according to the inclusion criteria, using the registrations of care at baseline, and submitted this list to the registered nurse or certified nurse assistant. Each subgroup was then assigned a single resident at the first meeting, whilst the steps of the intervention were being applied and practiced. In 5 meetings, the selected residents were assessed and treated, and the team of certified nursing assistants and/or registered nurses initiated and carried out the intervention i.e. incorporated the steps into their daily care.

#### Additional training for elderly care physicians

All elderly care physicians received an additional training from the expert physician (co-author WA) based on current guidelines for pain and behaviour issued by the Dutch Association of Elderly Care Physicians and Social Geriatricians (Verenso) [31,32], and the World Health Organisation [33].

### Data collection

Data were collected using a mixed-methods design (Table 2). This included qualitative data from: 1) notes and memos during the study period describing utilisation and feasibility of the intervention, details of the training, trainers and organisational changes; 2) semi-structured interviews with healthcare professionals focusing on how the intervention was implemented, and the influencing factors. The interviews were conducted by the first author (MP) and a research assistant (psychologist) and took place on-site using a topic list to structure the interview. Questions included: “What are your experiences working with STA OP!?”, “What facilitated (hindered) the application of STA OP!? and “How is the STA OP! intervention embedded on the unit, and in the nursing home?” The number of healthcare professionals, as well as the (re)presentation of disciplines, varied per interview. Due to organisational changes (understaffing and heavy workload) it was not possible to conduct an interview with healthcare professionals on 2 of the 11 units; 3) written evaluations by the trainers/instructors, concerning how the training was performed, and the trainer’s notes reflecting on the meetings; 4) completed forms of the STA OP! assessments performed, concerning reasons for starting/stopping the intervention and the intervention steps being applied; and 5) quantitative data on organisational changes/factors, and on the training and the manual, derived from questionnaires filled-out anonymously by the healthcare professionals and managers, and from the registrations of care (research database).

**Table 2:** Overview of data sources, sorted by organisational level.

Organisational level	Data source			Time of collection	Number of collections (N)

	Qualitative data	Combination qualitative and quantitative data	Quantitative data		

Level *Organisation/Management (unit/nursing home)*	Notes and memos of the coordinator and research assistant			On-going during study	
		Questionnaire for managers/management staff; written evaluations regarding organisational changes and factors		T2	12
Level *Multidisciplinary team*	Semi-structured interviews			T2	6
	Written evaluations by trainers/instructors			T1	4
	Notes and memos of the coordinator and research assistant			On-going during study	
		Questionnaire for healthcare professionals; written evaluations regarding the STA OP! training & training manual		T1	136
Level *Individual; Resident/Healthcare professional*	Completed forms of STA OP! assessments (residents)			On-going during study	58
			Registrations regarding care (research database)	T0–T1–T2	148

*Note*: Time of collection, T0 = baseline, T1 = 3 months and T2 = 6 months.

### Data analysis

Qualitative data from the interviews, written evaluations by healthcare professionals, management staff, trainers, and the notes of the coordinator and research assistant, were analysed according to the steps of thematic analysis [34].

Firstly, all interviews were digitally recorded, transcribed verbatim, checked, anonymised and re-read to increase familiarisation. Secondly, the semi-structured interviews were analysed independently by the project team (MP, AF, JT, WA) to increase the quality of the analyses and to create an initial list of codes together. At this stage, coding was performed openly and inductively, guided by themes directly derived from the text of the interviews. Thirdly, codes were merged, refined and sorted into a hierarchy of more abstract, overarching and sub-themes. Deviant codes and/or (sub)themes were discussed with the entire project team until consensus was reached and they had agreed on major themes. Lastly, the initial coding framework was used to analyse the written evaluations by healthcare professionals, management staff, trainers/instructors, and the notes/memos of the coordinator and research assistant. Qualitative data analysis was facilitated by ATLAS.ti software. Descriptive statistics and univariate analyses were used for the quantitative data of the written evaluations by healthcare professionals and management staff, and the research database, supported by IBM SPSS statistics version 22.0.

## Results

### Experiences of health care professionals

From the written evaluations of healthcare professionals and the semi-structured interviews, it appeared that the manual, the training, as well as the steps of the intervention were found to be very informative, relevant and feasible by all healthcare professionals.

Nurse:

“I found it all very clear. It’s written down as clearly as daylight – so that you can elaborate on each step without needing any explanation or clarification.”

Additionally, the evaluations and interviews showed that the non-pharmacological steps were valued most; due to the bottom-up organisation of the intervention the nursing staff was ‘in the lead’. They could make a difference themselves, independently from third parties, which made them feel empowered and motivated.

The interviews also indicated that the training was intensive. However, the written evaluations indicate that only 29.4% of the 136 participants found the meetings to be too long, and 12.5% indicated that they contained too much information. Healthcare professionals rated (maximum 10) the multidisciplinary team training as (on average) 7.6 (SD = 0.94), and the manual and accompanying forms also as 7.6 (SD = 1.04). In general, the ambience was pleasant during the meetings; participants felt comfortable with the trainer (97.1%) and their colleagues (96.3%). In addition, 94.0% was (very) satisfied with the knowledge and skills of the trainer concerning the content of dementia, pain and challenging behaviour, as well as the motivation/involvement of the individuals, and the group as a whole. A total of 136 healthcare professionals received a certificate (all except for 8 who missed more than one of five training sessions; reach, 94.4%).

### Delivery and implementation of the intervention

#### Multidisciplinary intervention; planned disciplines, meetings and steps

On most of the units (8/11, reach 72.7%), the predefined disciplines were present during the meetings: on 2/8 units the whole multidisciplinary team was present during all the meetings due to the importance that management attached to efficient/structured interdisciplinary learning and cooperation. However, on 3/11 units, apart from the nursing staff and a psychologist, no other disciplines attended the meetings due to structural or incidental problems at the organisational level. On 10/11 units all 5 planned meetings took place (dose 95.4%), the necessary feedback and communication skills were trained, and the STA OP! assessment was carried out; on 1 unit the final meeting of one of the groups was planned twice, but could not take place due to organisational difficulties (i.e. understaffing/no facilitation by management).

#### Selection of residents at the first meeting

In total, 130/148 residents (reach 87.8%) met the predefined inclusion criteria and were eligible for treatment with STA OP! A total of 58 residents (dose 39.2%) were assessed and treated with the STA OP! intervention. The teams selected of these residents pragmatically because the most foreseeable, predominant or stressful behaviours, for the residents themselves or for the healthcare professionals in general (source completed forms and research database). In addition, for 48/58 residents (82.8%), the completed forms showed that challenging behaviours (e.g. agitation/aggression, verbalisations, and resistance to care) were the main reason for starting the STA OP! intervention. In 2/58 residents (3.4%), pain was mentioned as the main reason, and in 8/58 residents (13.8%) this was a combination of pain and challenging behaviour.

#### Additional training for elderly care physicians

Besides the multidisciplinary team training, all involved elderly care physicians (n = 7 participated in the intervention group) attended the additional training on management of pain in patients with dementia. The additional training was based on current national and international guidelines for pain and behaviour [31,32].

### Facilitating and impeding factors in the implementation process

Factors playing a role in the implementation process were mainly on the organisational/management and team level; these interacted with each other, as well as with those that played a role in the application on the resident/professional level. Themes that emerged of the interviews were ‘Intervention and Training with sub-themes workload, content, and usability’, ‘Implementation with sub-theme securing the intervention’, ‘Empowerment of staff’, ‘Leadership’, Interdisciplinary learning and cooperation’, and ‘Organisational factors with sub-themes staff overturn, shortage of staff and management’. The specific facilitating/impeding factors are described below.

### Facilitating and impeding factors associated with the level of the organisation

#### Organisational changes or other innovations at the time of the implementation impeded the implementation process

Notes and memos of the research assistant, and written evaluations of the management staff indicated that despite the agreement at the start, some units became involved in various other projects besides the implementation of STA OP!, e.g. implementing and using new forms for quality improvement on the units, or implementing electronic patient files. This overload of new information and methods made it difficult for the teams to focus on implementing the STA OP! intervention and impeded the implementation process.

#### Staff turnover, shortage of staff and high workload affected the multidisciplinary nature and continuity of the intervention

During the implementation process, some units encountered structural problems at the level of the organisation (source notes and memos of the research assistant and coordinator): staff turnover or absence of essential disciplines and/or nursing staff (shortage of staff) affected the multidisciplinary nature and continuity of the intervention. At times, physicians not being much present or communicative obstructed the process. As a consequence, these organisational problems caused high workload and were mentioned as impeding factors for implementation.

### Facilitating and impeding factors associated with the level of the team

#### Presence of a person with a motivational leadership style facilitated implementation

Interviews and written evaluations of healthcare professionals indicated that a (key) person with a stimulating and motivational leadership style was a facilitating factor for implementation; most often female, respected, motivated and involved professionals fulfilled this position. They were enthusiastic, open to change, encouraged healthcare professionals to use the intervention, created support and put organisational matters in order; 9 of the 11 motivational leaders at the units attended all the meetings of the team training. Absence of such a leader on a unit impeded implementation.

##### Interdisciplinary learning and cooperation; facilitated by the design of the training, but in clinical practice hindered by the composition of the pre-planned subgroups

At least 80% of the healthcare professionals participated in the multidisciplinary training, and the subgroups formed for educational purposes consisted of various disciplines, which created a motivating and stimulating climate during the meetings. To retain a minimum of staff on the work floor, most of the training sessions were offered twice and scheduled for two groups of healthcare professionals. Designing the training in this way proved to facilitate interdisciplinary learning and cooperation. In addition, evaluations of the trainers indicated that a relatively large amount of time was spent on giving feedback, mutual cooperation/collaboration, and communication to facilitate this process.

Written evaluations by healthcare professionals, trainers, and the semi-structured interviews, indicated that the multidisciplinary character of the intervention and the diversity of the subgroups was highly valued.

Healthcare professionals gained insight into each other’s expertise and, as a consequence, disciplines were able to apply their expertise better and more specifically; they found it easier to contact each and at an earlier stage, i.e. they were easily accessible when questions arose on the treatment of challenging behaviours, or ambiguities occurred concerning the application of certain steps. Not only did disciplines learn from each other when problems/questions emerged during the meetings, but they also learned for future residents in similar situations on-the-job.

Nurse assistant:

“What I really liked was the fact that we were participating in this training as a whole multidisciplinary team including all related disciplines, not only as a single nursing team. For example, a psychologist looks at problems in a different way, i.e. from another point of view. I thought: I’ve never really seen it that way – but I guess you’re right.” ……… “I think it contributed to the fact that the barriers for contacting the other disciplines have become smaller, they’re more easily accessible now.”

Psychologist:

“The nursing staff has a lot of fun in finding out why someone behaves in a certain way. Now, they ask me at an earlier stage how to deal with challenging behaviours, and as such I can do my job better, more targeted, and with more members of the team.”

However, the interviews also showed that some of the pre-planned subgroups of healthcare professionals turned out to be suboptimal in clinical practice (on-the-job). Due to the composition of the subgroups, practical or logistical problems with regard to collaboration and consultation of the subgroup members occurred, amongst mutual and/or different discipline(s); i.e. different shifts or working days and not being able to meet one another. Creativity and flexibility regarding these problems differed between the subgroups and units; some subgroups spent time outside working hours, whereas in other cases the group fell apart, resulting in delayed assessment of residents and mastery of the different steps. Clear agreements and written reporting or transfers facilitated the process of interdisciplinary cooperation.

Registered nurse:

“The hardest thing was working together on-the-job in subgroups, which consisted of different disciplines. Since we all had different schedules and days off, but at the same time had to assess the steps in groups, someone took the lead and then others took over if we had only a short time together to fill-out the forms. That’s how we solved it.”

In addition, the absence of disciplines due to impeding factors at the organisational level affected the multidisciplinary character of the intervention, as well as interdisciplinary cooperation; in some cases essential parts of the intervention could not be performed at all, or only at a much later stage, which impeded implementation at these units.

##### Securing the intervention to regularly used meetings and structures stimulated the utilisation of the intervention

By securing the intervention in the patient file, regular (team) meetings and internal structures (like an internal educational academy) utilisation was stimulated. Moreover, the intervention became visible and was discussed more frequently, resulting in improved awareness among healthcare professionals, and facilitated interdisciplinary cooperation as well as implementation, (source written evaluations by healthcare professionals and interviews).

##### Formation of core teams at the end of the training period was suboptimal, due to logistical problems

At 10/11 units the core teams were formed at the last meeting (dose, 90.1%). Interviews and written evaluations of healthcare professionals indicated that forming those teams at the end of the training period proved to be suboptimal; most teams had a problem getting together when the structured meetings of the multidisciplinary team training had ended (fidelity, 50%). On units where these difficulties did not exist, the core team acted as a coach and facilitator during implementation.

### Facilitating and impeding factors associated with the level of the individual resident/professional

#### Systematically observing behaviours and the STA OP! assessment seen as surplus value

The stepwise working method (i.e. systematically observing behaviours and the STA OP! assessment) is seen as a surplus value in substantiating treatments.

Nurse:

“It’s actually easier now to try out pain medication. Elderly care physicians were often reluctant – but with this stepwise intervention we have more evidence to support our request for treatment.”

In addition, healthcare professionals became more aware of pain as a cause of challenging behaviour, the effects of their own actions, and of the unmet needs of the residents. Seeing results motivated them to utilise the intervention and acted as facilitating factor for implementation.

Nurse:

“Well, the moment of getting her out of bed was always… how shall I say…. Well, most of the time we thought: we’ll help her after our coffee break, around 11 o’clock–11.30. But then I noticed, when we helped her to get out of bed, say, around 8 o’clock–8.30, that she came singing out of bed, went to breakfast, and was quite relaxed.”

#### Steps of the intervention seemed insufficient in acute or palliative phases

In contrast, the interviews showed that the steps did not seem immediately useful in acute situations or in a palliative phase; steps were skipped mainly due to time constraints, resulting in eliminating the systematic element of the intervention.

Nurse:

*“In practice you sometimes notice that steps are passed over in acute situations, because it just works that way … for example, if someone suddenly becomes very confused or rapidly deteriorates physically”*.

## Discussion

The aim of this study was to describe the implementation process of the STA OP! intervention, i.e. 1) to gain insight into the healthcare professionals’ experiences regarding implementation of the intervention and its usage in daily practice, 2) to examine the extent to which the STA OP! intervention was delivered and implemented as intended, at the level of the team and the individual resident/professional, and 3) to understand factors influencing the implementation process.

From the perspective of the healthcare professionals, the stepwise intervention provided a useful structure for the delivery of dementia care in residents with pain and challenging behaviour. Moreover, healthcare professionals stated that it created or increased awareness of pain as a cause for challenging behaviour, and empowered them. Furthermore, this process evaluation showed that a motivational leader facilitates implementation. Earlier, Kovach et al. (2012) reported that a person with motivational leadership skills (who acts as a coach/facilitator) also secures the forms and intervention to regular team meetings and/or structures, and is crucial during the implementation period [35].

This process evaluation also shows that the intervention was not always implemented and actually used as planned on all units. Impeding factors were mainly found on the level of the team. Individual motivation and capability factors have been identified as major factors affecting implementation processes, in addition to social processes and environmental factors [8].

In line with other studies [25,26,27,28,36,37], staff turnover, high workload, concurrent projects, and organisational changes were described as barriers for implementing the intervention. In addition, we found that the absence of pre-defined disciplines during the training sessions was a barrier for implementation; on some units only (part of) the nursing staff attended the training sessions, moreover, other pre-defined disciplines were absent. This affected the multidisciplinary character of the intervention and training sessions, which led to impaired or absent interdisciplinary learning, cooperation and communication and, eventually, to suboptimal implementation. Also, Simpson et al., described that, in the USA, engaged staff and educational reinforcement were essential elements for successful implementation of the STI [38]. In addition, in the Netherlands, nursing homes employ elderly care physicians that have an officially recognized 3-year training for working with complex problems of elderly in long-term care [39]. Although we found that this often proved to be a facilitator in the implementation process, when the physician was absent or communication with him/her was difficult, it proved to obstruct the process. This lack of physician collaboration was also found in the process evaluation of the STI in the USA [38].

Despite that implementing a complex intervention in the context of a long-term care setting remains challenging [40,41,42], the present process evaluation revealed modifiable factors that enhance and facilitate implementation, resulting in the following recommendations for future implementation:

At the level of the organisation:

- Commitment and facilitation by the management; providing stability (i.e. no other innovations/changes at the same time), support and a shared focus to change, are essential elements for a proper implementation. If these conditions cannot be met, first, efforts have to be made to create better conditions.

At the level of the team:

- Implementation should, preferably, start on units with a motivational leader: a person who is enthusiastic, respected, open to change, well-acquainted with the content through active involvement in the training, and who can motivate and stimulate professionals in the utilisation and implementation of the intervention. If such a motivational leader is not available, then efforts must be made to find a person within the multidisciplinary team who is willing/capable to take on this position.
- Involve and engage a whole multidisciplinary team of healthcare professionals, by facilitating participation in the training (preferably in all the meetings), to facilitate interdisciplinary learning, mutual collaboration/cooperation and communication.
- Create and initiate a core team of healthcare professionals at the beginning of the training (i.e. the first meeting) in order for them to act as a coach and facilitator during the whole training and implementation period.
